# Securing IoT-Based RFID Systems: A Robust Authentication Protocol Using Symmetric Cryptography

**DOI:** 10.3390/s19214752

**Published:** 2019-11-01

**Authors:** Khwaja Mansoor, Anwar Ghani, Shehzad Ashraf Chaudhry, Shahaboddin Shamshirband, Shahbaz Ahmed Khan Ghayyur, Amir Mosavi

**Affiliations:** 1 Department of Computer Science, Air University Islamabad, Islamabad 44000, Pakistan; kh.mansoorulhassan@gmail.com; 2 Department of Computer Science & Software Engineering, International Islamic University Islamabad, Islamabad 44000, Pakistan; anwar.ghani@iiu.edu.pk (A.G.); shahbaz.ahmed@iiu.edu.pk (S.A.K.G.); 3 Department of Computer Engineering, Faculty of Engineering and Architecture, Istanbul Gelisim University, Istanbul 34310, Turkey; Shahzad@iiu.edu.pk; 4 Department for Management of Science and Technology Development, Ton Duc Thang University, Ho Chi Minh City, Viet Nam; 5 Faculty of Information Technology, Ton Duc Thang University, Ho Chi Minh City, Viet Nam; 6 Faculty of Health, Queensland University of Technology, Victoria Park Road, Kelvin Grove, QLD 4059, Australia; amir.mosavi@kvk.uni-obuda.hu; 7 Kando Kalman Faculty of Electrical Engineering, Obuda University, 1034 Budapest, Hungary

**Keywords:** authentication protocol, IoT Security, RFID security, symmetric cryptography

## Abstract

Despite the many conveniences of Radio Frequency Identification (RFID) systems, the underlying open architecture for communication between the RFID devices may lead to various security threats. Recently, many solutions were proposed to secure RFID systems and many such systems are based on only lightweight primitives, including symmetric encryption, hash functions, and exclusive *OR* operation. Many solutions based on only lightweight primitives were proved insecure, whereas, due to resource-constrained nature of RFID devices, the public key-based cryptographic solutions are unenviable for RFID systems. Very recently, Gope and Hwang proposed an authentication protocol for RFID systems based on only lightweight primitives and claimed their protocol can withstand all known attacks. However, as per the analysis in this article, their protocol is infeasible and is vulnerable to collision, denial-of-service (DoS), and stolen verifier attacks. This article then presents an improved realistic and lightweight authentication protocol to ensure protection against known attacks. The security of the proposed protocol is formally analyzed using Burrows Abadi-Needham (BAN) logic and under the attack model of automated security verification tool ProVerif. Moreover, the security features are also well analyzed, although informally. The proposed protocol outperforms the competing protocols in terms of security.

## 1. Introduction

**Figure 1:**
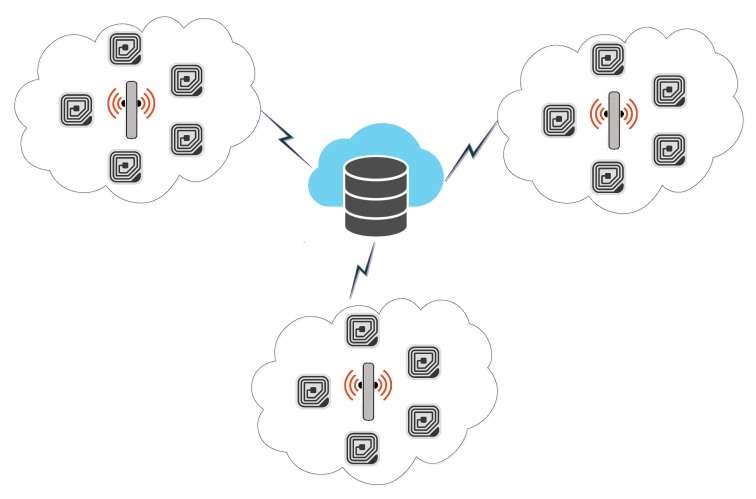
Radio Frequency Identification (RFID) System Architecture.

Since its inception, the Internet of Things (IoT) is an emerging idea and is defined as, “A system of interrelated computing devices, mechanical and digital machines, objects, animals, or people that are provided with unique identifiers (UIDs) and the ability to transfer data over a network without requiring human-to-human or human-to-computer interaction” [1]. The devices are equipped with internet and are capable of communicating with other devices, and such systems are administered and monitored remotely [2,3]. The IoT assimilates heterogeneity of networks, such as smart cities, sensor networks, smart grids, Radio Frequency Identification (RFID), and transportation and parking systems. The RFID is also on its way to replace conventional bar code systems, as the latter have limitations, including line of sight communication, very limited storage capacity, and prone to physical damage. The overall RFID system architecture is depicted in Figure 1.

RFID is simplest form of pervasive sensor networks and is commonly used for identification of physical objects [4,5]. Systems based on RFID consist of a tag, which is equipped with a transceiver to send and receive radio signals from connected devices [6,7]. The RFID Reader is the other device which acts as an access point and can receive and send messages to transceivers. The Reader is also responsible for the availability of tag information at application level [8,9,10]. RFID tags can be passive, as well as active. Table 1 summarizes the features of passive and active tags.

RFID systems are typically used for object tracking and identification purposes. The system application accessed through Reader can perform data processing for onward usage in a range of applications like: Asset Tracking, Race Timing, E-Passport, Transportation, Payments, Human Implants, Supply-Chain-Management, Fleet and Asset-Management, Security Access-Control, E-Commerce, and Traffic Analysis and Management [9,11,12,13,14]. The IoT-enabled RFID system facilitates all such systems without any physical exposure and in bulk. However, such facilities come with security threats because of the underlying wireless media used for the communication between the tag and the Reader [15,16,17]. To make an RFID system acceptable and meet industrial standards, the following security features should be considered during the design phase of RFID security schemes:

1. The security scheme should preserve user privacy and anonymity.
2. The scheme should ensure forward and backward secrecy.
3. The scheme should prevent insider attacks and replay attacks.
4. The system should have capabilities to withstand impersonation and forgery attacks.
5. The system should provide mutual authentication and thwart man in middle attack.
6. The system should be user-friendly and should have the provision of updation and alteration of tag data at any time.

Various authentication protocols have been proposed for securing RFID systems [3,12,13,17,18,19,20,21,22,23,24,25,26,27,28,29,30,31,32,33]. Some of these protocols are based on public key infrastructure (PKI) [12,18,19,32,33]. Due to the resource-constrained nature of RFID, the protocols based on PKI are unenviable. Some of the schemes have been proposed on lightweight cryptographic primitives. However, many such schemes based on merely the lightweight primitives were proved as insecure [23,27,29,30,31].

In 2005, Yang et al. proposed an RFID authentication protocol based on only exclusive-OR (XOR) and hash functions [23]. Some other protocols were also proposed in [21,26,30], using only lightweight hash, XOR and/or symmetric encryption. Despite their [21,23,26,30] claims to provide flawless security, Piramuthu [28] proved that the protocols in [23,30] are vulnerable to replay attack, the protocol in [26] is vulnerable to impersonation of the tag and the Reader, and protocol proposed by Cai et al. [30] is vulnerable to denial-of-service (DoS) and impersonation of tag. Cho et al. [31] then proposed another hash-based protocol for securing RFID. However, Safkhani et al. [29], through cryptanalysis, proved that Cho et al.’s protocol is insecure against DoS, as well as impersonation attacks. Another authentication protocol for securing RFID systems using only symmetric key operations was proposed by Ayaz et al. [17]. However, in their protocol [17], the authentication is performed on the basis of biometrics verification. Such biometric verification may not be desirable in many scenarios, like anti-counterfeiting of life saving drugs, recording and counting number of specific goods moving in and out of a store, etc.

### 1.1. Motivations and Contributions

Quite recently, Gope and Hwang [3] argued that the existing protocols [21,23,26,29,30,31] based on hash functions are impractical. Then Gope and Hwang presented a new lightweight authentication protocol using only hash functions. They claimed to avoid all known attacks while maintaining efficiency. However, in this paper, we show that the protocol of Gope and Hwang is vulnerable to collision, DoS, and stolen verifier attacks. Moreover, this article presents an improved and robust protocol using only lightweight symmetric cryptography primitives for IoT-based RFID systems to resist all known attacks. The general contributions of this article include:

- Cryptanalysis of the baseline [3] protocol.
- Proposed an improved authentication protocol using only lightweight symmetric key primitives to overcome the security issues of the baseline protocol.
- Performed formal and informally security analysis of the proposed protocol.
- Solicited the comparison of the proposed protocol with related existing protocols with respect to security features.
- Accomplished the comparison of the proposed protocol with related existing protocols with respect to performance, including communication, as well as computation complexity.

### 1.2. Adversarial Model

The proposed protocol is designed keeping in mind the following adversarial model where common assumptions as pointed out in [34] are made. The following assumptions are considered as the capabilities of the adversary A.

1. The public channel is under full control of A, so that the A can intercept, revert, modify, replay, or even send a fresh fabricated message.
2. A has the capability to extract some of the information of the tag by power analysis. However, shared key of the tag and Server is secret and is inaccessible to any adversary.
3. A can be any deceitful tag or an outsider of the system.
4. The database attached to the Server is inaccessible, and no adversary A can access the private key of the Server.

### 1.3. Road Map

The rest of the article is organized in various sections. In Section 1.4, a brief overview of the protocol of Gope and Hwang [3] is presented. Section 2 presents the proposed protocol, whereas Section 3 presents the detail security analysis of the proposed protocol. Section 4 presents the comparative analysis of the proposed protocol with existing protocols, and, finally, Section 5 concludes the article.

### 1.4. Review of Baseline Protocol

This section first reviews the baseline protocol of Gope and Hwang’s [3] and then performs its cryptanalysis. Table 2 presents some of the notations used in the baseline protocol. The proposed protocol designed for RFID consists three main entities: (1) Database Server, (2) Reader Device, and, (3) RFID tags. The network layout of the RFID System divided into several RFID clusters. Every cluster consists of a Reader and many tags. Tags can shift from one cluster to another. Every Reader of the cluster authenticates the registered tags through the Database Server. Each Reader and Database Server share a symmetric key $K_{rs}$ [3]. Gope and Hwang’s [3] authentication scheme consists of two main phases: (1) tag Registration Phase and (2) tag Authentication Phase.

### 1.5. Baseline Protocol Tag Registration Phase

**Figure 2:**
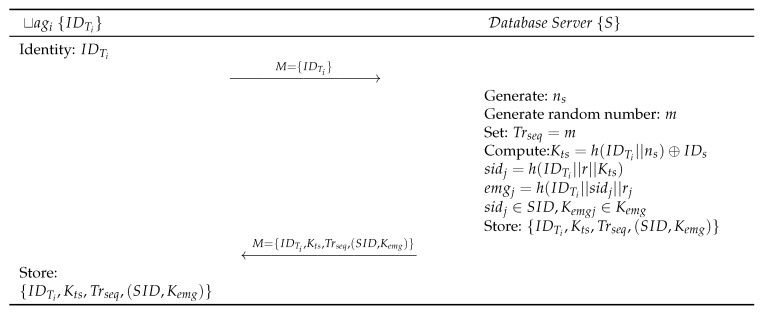
Gope-Hwang’s proposed registration scheme.

The following steps, as shown in Figure 2, are performed for tag registration:

Step BLR 1: ${\mathrm{tag}}_{i}\overset{{\mathrm{ID}}_{T_{i}}}{⟶}S$
  Each tag ($tag_{i}$) submits $ID_{T_{i}}$ to the Server *S*.
Step BLR 2: $S\overset{M}{⟶}{\mathrm{tag}}_{i}:\langle M=\{K_{\mathrm{ts}},\left(\mathrm{SID},K_{\mathrm{emg}}\right),{\mathrm{Tr}}_{\mathrm{seq}},h\left(.\right)\}\rangle$
  *S* generates random number $n_{s}$ and computes $K_{ts}=h\left(ID_{T_{i}}∥n_{s}⊕ID_{s}\right)$. *S* then generates a set of unlikeable shadow identities $ID_{s}$, and $SID=\{sid_{1},sid_{2}\cdots \}$, where the $sid_{j}\in SID$. *S* computes $sid_{j}=h(ID_{T_{i}}∥r_{j}∥K_{ts})$. Further, *S* generates a set of emergency keys $K_{emg}=\left\{k_{emg_{1}},k_{emg_{2}}\cdots \right\}$, each of the keys corresponding to specific $sid_{j}\in SID$, where each $k_{emg_{i}}\in K_{emg}$. *S* then computes $k_{emg_{i}}=h(ID_{T_{i}}∥sid_{j}∥r_{j})$. Then *S* generates a 32-bit random sequence number $Tr_{seq}$ and random number *m* and matches it with $Tr_{seq}$, $Tr_{seq}=m$. *S* then sends the $Tr_{seq}$ to the $tag_{i}$ through Reader $R_{i}$ by maintaining the copy of $Tr_{seq}$ in its database for speeding up the authentication process. *S* authenticates the validity of RFID tag $ID_{T_{i}}$ based on $TR_{seq}$. If $TR_{seq}$ does not have a match within the record of *S*, it terminates the process. In this case, the RFID tag $ID_{T_{i}}$ will use one of its fresh pair of the emergency key $k_{emg_{j}}\in K_{emg}$ and shadow ID $sid_{j}\in SID$. The used pair of shadow ID and emergency ID ($SID,K_{emg}$) must be deleted from both, the Database Server *S* and the RFID tag $ID_{t_{i}}$. Database Server *S* again updates and send $\left\{K_{ts},\left(SID,K_{emg}\right),Tr_{seq},h\left(.\right)\right\}$ through a secure channel for further communication.
Step BLR 3: $tag_{i}$, upon receiving message from *S*, stores $\left\{ID_{T_{i}},K_{ts},\left(SID,K_{emg}\right),Tr_{seq},h\left(.\right)\right\}$ in its memory.

### 1.6. Baseline Protocol Tag Authentication Phase

**Figure 3:**
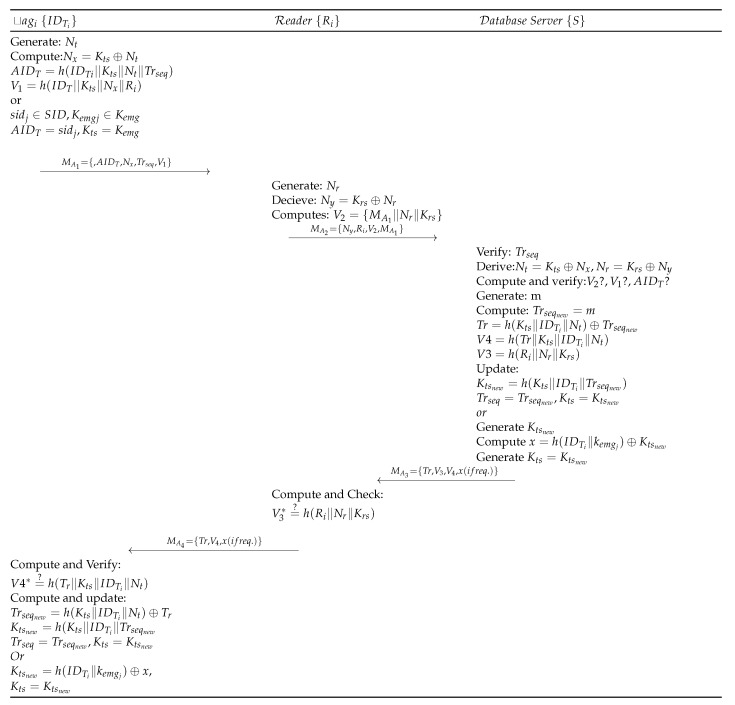
Gope-Hwang’s proposed authentication scheme.

The registered tag initiates the authentication process, as shown in Figure 3, and is detailed as follows:

Step BLA 1: ${\mathrm{tag}}_{i}\overset{M_{A_{1}}}{⟶}R_{i}:\langle M_{A_{1}}=\left\{{\mathrm{AID}}_{T},N_{x},{\mathrm{Tr}}_{\mathrm{seq}},V_{1}\right\}\rangle$
  $tag_{i}$ with identifier $ID_{T_{i}}$ generates random number $N_{t}$, and derives $AID_{T}=h(ID_{T_{i}}∥K_{ts}∥N_{t}∥Tr_{seq})$, $N_{x}=K_{ts}⊕N_{t}$. The tag then computes $V_{1}=h(AID_{ti}∥K_{ts}∥N_{x}∥R_{i})$ and sends message request as $M_{A_{1}}$ to the Reader device $R_{i}$. $R_{i}$ also receives a recently used sequence number from *S* for mutual authentication. In the case of synchronization loss, the tag uses one of its fresh pair $\left(sid_{j},K_{emg_{j}}\right)$. Subsequently, it is assigned to the $sid_{j}$ as $AID_{T}$ and then $k_{emg_{j}}$ as $K_{ts}$. $tag_{i}$ sends $M_{A_{1}}$ to the Reader $R_{i}$.
Step BLA 2: $R_{i}\overset{M_{A_{2}}}{⟶}S:\langle M_{A_{2}}=\left\{N_{y},R_{i},V_{2},M_{A_{1}}\right\}\rangle$
  Upon receiving request from $tag_{i}$, Reader $R_{i}$ of the $i^{th}$ cluster (in which $tag_{i}$ is located) generates random number $N_{r}$ and computes $N_{y}=K_{rs}⊕N_{r}$, $V_{2}=h(M_{A_{1}}∥N_{r}∥K_{rs})$. $R_{i}$ then sends $M_{A_{2}}$ to *S* for verification.
Step BLA 3: $S\overset{M_{A_{3}}}{⟶}R_{i}:\langle M_{A_{3}}=\left\{T_{r},V_{3},V_{4},x_{\left(\mathrm{if}\mathrm{req}.\right)}\right\}\rangle$
  When *S* receives a request from $R_{i}$, first it validates the track sequence number $Tr_{seq}$ by computing $V_{1}=h(AID_{T}∥K_{ts}∥N_{x}∥R_{i})$. *S* then derives $N_{t}=K_{ts}⊕N_{x}$ and verifies $AID_{T}$. Upon successful verification of $AID_{T}$, *S* generates a random number *m* and assigns it to $T_{r_{seq}}=m$. *S* also computes $T_{r}=h(K_{ts}∥ID_{T_{i}}∥N_{t})⊕T_{r_{seq}}$, $V_{4}=h(T_{r}∥K_{ts}∥ID_{T_{i}}∥N_{t})$, $V_{3}=h(R_{i}∥N_{r}∥K_{rs})$ to create a message $M_{A_{3}}$ and the *S* sends $M_{A_{3}}$ to $R_{i}$. Finally, *S* computes $K_{Ts_{new}}=h(K_{ts}∥ID_{T_{i}}∥Tr_{seq_{new}})$ and updates $K_{Ts_{new}}$ and $Tr_{seq_{new}}$. In case the message $M_{A_{1}}$ does not contain $T_{r_{seq}}$, then *S* randomly generates a new shared key $K_{TS_{new}}$ using the emergency key $K_{emg_{j}}$ and real identity of the tag $ID_{T_{i}}$. Then $x=K_{ts_{new}}⊕h\left(ID_{T_{i}}∥K_{emg_{j}}\right)$ is computed and *x* is sent with the message $M_{A_{3}}$, where $V_{4}$ is calculated as $V_{4}=h(N_{t}∥T_{r}∥x∥K_{emg_{j}})$.
Step BLA 4: $R_{i}\overset{M_{A_{4}}}{⟶}{\mathrm{tag}}_{i}:\langle M_{A_{4}}=\left\{T_{r},V_{4},x_{\left(\mathrm{if}\mathrm{req}.\right)}\right\}\rangle$
  $R_{i}$ receives $M_{A_{3}}$ and computes $h(R_{i}∥N_{r}∥K_{rs})$, and validates if it is equal to $V_{3}$. Upon successful validation, $R_{i}$ sends $M_{A_{4}}$ to $tag_{i}$. Contrarily, the Reader $R_{i}$ terminates the session.
Step BLA 5: $tag_{i}$, on receiving $M_{A_{4}}$, computes $h(Tr∥K_{ts}∥ID_{T_{i}}∥N_{t})$ and verifies its equality with $V_{4}$. Upon success, $tag_{i}$ derives $K_{ts_{new}}=h(K_{ts}∥ID_{T_{i}}∥Tr_{seq_{new}})$ and stores $K_{ts}=K_{ts_{new}}$, $Tr_{seq}=Tr_{seq_{new}}$ for future communication.

### 1.7. Cryptanalysis of Baseline Protocol

The following subsections show that the baseline protocol is vulnerable to: (1) Collision, (2) Stolen Verifier, and (3) DoS Attacks.

#### 1.7.1. Vulnerable to Collision Attack

The correctness of the baseline protocol [3] depends on Track sequence number $Tr_{seq}$, generated randomly during registration and saved in the database, as well as in the tag’s memory. This number $Tr_{seq}$ is sent in authentication request $M_{A_{1}}=\left\{AID_{T},N_{x},Tr_{seq},V_{1}\right\}$ by the tag and then, upon reception of $M_{A_{1}}$, the Reader $R_{i}$ sends $M_{A_{2}}=\left\{M_{A_{1}},N_{y},R_{i},V_{2}\right\}$ to the Database Server. *S* verifies the legitimacy of $Tr_{seq}$ by comparing it with the one stored in its database. The randomness can cause two or more track sequence numbers to have the same value (collision), and there is no mechanism to handle such collisions. Then the process will terminate abnormally, and the legitimate tag will be deprived of its right to authentication and access.

#### 1.7.2. Vulnerable to Stolen Verifier Attack

Considering the common adversarial modal, as mentioned in Section 1.2, an adversary can steal the verifier table stored unencrypted on server. A based on the track sequence number $Tr_{seq}$ and the public request from any of the previous session $M_{A_{1}}:\left\{AID_{T},N_{x},Tr_{seq},V_{1}\right\}$ and $M_{A_{2}}:\left\{N_{y},R_{i},V_{2},M_{A_{1}}\right\}$ can then generate login request using the previous session’s $N_{x}$, $N_{y}$ and the stolen new $Tr_{seq}$. The request will pass the authentication test as all values are valid. Hence baseline protocol is also vulnerable to stolen verifier attack.

#### 1.7.3. Vulnerable to DoS Attack

In the baseline proposal [3], an adversary can launch a DoS attack by continuously generating 32 bits random $Tr_{seq}$ numbers and send it to the Database Server. It will keep *S* busy in verifying dummy random numbers, thus restricting *S* to serve a legitimate request.

## 2. Proposed Scheme

Like the baseline protocol, the proposed protocol for RFID consists of three main entities: (1) Database Server, (2) Reader Device, and (3) RFID tags. The network layout of the RFID System is divided into several RFID clusters. Every cluster consists of a Reader and many tags. Tags can shift from one cluster to another. Every Reader of the cluster authenticates the registered tags through the Database Server. Each Reader and Database Server share a symmetric key $K_{rs}$. Proposed improved authentication scheme consists of two main phases; (1) tag Registration Phase, (2) tags Authentication Phase.

### 2.1. Tags Registration Phase

**Figure 4:**
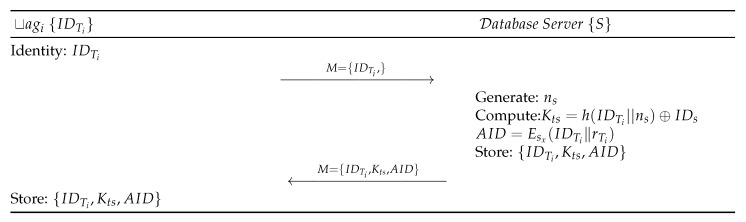
Registration phase of the proposed protocol.

The following steps, as shown in Figure 4, are performed for tag registration:

Step PTR 1: ${\mathrm{tag}}_{i}\overset{{\mathrm{ID}}_{T_{i}}}{⟶}S$
  Each tag submits $ID_{T_{i}}$ to the Server *S*.
Step PTR 2: $S\overset{M}{⟶}{\mathrm{tag}}_{i}:\langle M=\{{\mathrm{ID}}_{T_{i}},K_{\mathrm{ts}},\mathrm{AID}\}\rangle$
  *S* generates a random number $n_{s}$ and computes $K_{ts}=h\left(ID_{T_{i}}∥n_{s}⊕ID_{s}\right)$. *S* generates $r_{i}$ randomly and computes one-time alias $tag_{i}$’s identity $AID=E_{s_{x}}\left(ID_{T_{i}}∥r_{T_{i}}\right)$ by encrypting it with the Secret Key $s_{x}$ of *S*. *S* authenticates $tag_{i}$ based on $AID_{T}$ in authentication phase by checking if a request is valid or not. *S* stores and sends *M* to the RFID tag through a secure channel.
Step PTR 3: Upon receiving the message from *S*, $tag_{i}$ stores the information $M=\{ID_{T_{i}},K_{ts},AID\}$ in its memory.

### 2.2. Tags Authentication Phase

**Figure 5:**
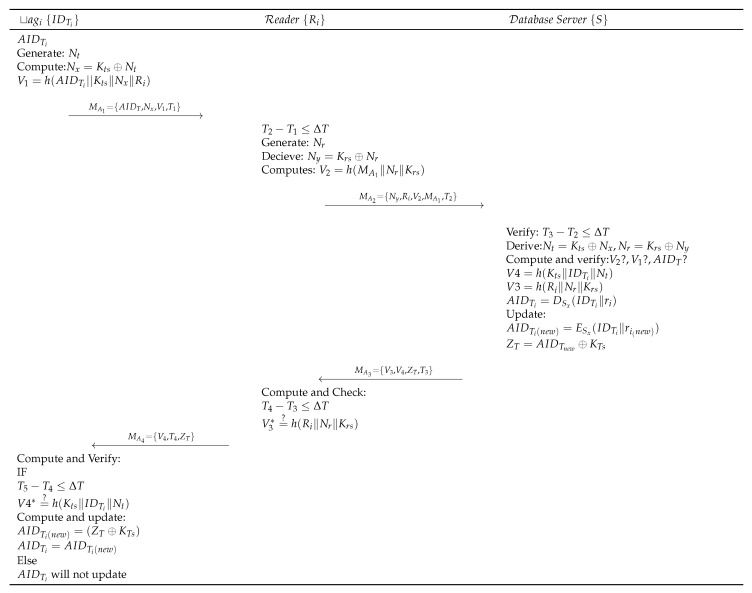
Proposed authentication protocol.

The registered tag initiates the authentication process, as shown in Figure 5, and is detailed as follows:

Step PTA 1: ${\mathrm{tag}}_{i}\overset{M_{A_{1}}}{⟶}R_{i}:\langle M_{A_{1}}=\left\{{\mathrm{AID}}_{T},N_{x},T_{1},V_{1}\right\}\rangle$
  RFID tag with identifier $ID_{T_{i}}$ generates a random number $N_{t}$ and derives $N_{x}=K_{ts}⊕N_{t}$ and $V_{1}=h(AID_{t}∥K_{ts}∥N_{x}∥R_{i})$. The tag then initiates an authentication request request by sending $M_{A_{1}}$ to $R_{i}$.
Step PTA 2: $R_{i}\overset{M_{A_{2}}}{⟶}S:\langle M_{A_{2}}=\left\{N_{y},R_{i},V_{2},M_{A_{1}},T_{2}\right\}\rangle$
  Upon receiving the request from the tag, Reader $R_{i}$ of the $i^{th}$ cluster (in which tag is located) first verifies the timestamp freshness as $(T_{2}-T_{1})\leq \Delta T$. $R_{i}$ generates a random number $N_{r}$ and computes $N_{y}=K_{rs}⊕N_{r}$, $V_{2}=h(M_{A_{1}}∥N_{r}∥K_{rs}∥T_{2})$. $R_{i}$ sends $M_{A_{2}}$ to the *S* for verification.
Step PTA 3: $S\overset{M_{A_{3}}}{⟶}R_{i}:\langle M_{A_{3}}=\left\{V_{3},V_{4},Z_{T},T_{3}\right\}\rangle$
  When *S* receives the request from $R_{i}$, first it verifies $(T_{3}-T_{2})\leq \Delta T$, then derives $N_{t}=K_{ts}⊕N_{x}$ and $N_{r}=K_{rs}⊕N_{y}$. Further, *S* computes and verifies $V_{1}=h(AID_{T}∥K_{ts}∥N_{x}∥R_{i})$, $V_{2}=h(M_{A_{1}}∥N_{r}∥K_{rs}∥T_{2})$. Then *S* verifies $AID_{T_{i}}$ by decrypting it as $AID_{T_{i}}=D_{S_{x}}\left(ID_{T_{i}}∥r_{i}\right)$. Upon successful verification, *S* computes $V3=h(R_{i}∥N_{r}∥K_{rs}∥T_{3})$ and $V4=h(K_{ts}∥ID_{T_{i}}∥N_{t}∥T_{3})$. *S* then updates $AID_{T_{i}\left(new\right)}=E_{S_{x}}\left(ID_{T_{i}}∥r_{i_{(}new)}\right)$ and computes $Z_{T}=AID_{T_{new}}⊕K_{Ts}$. *S*, finally, sends $M_{A_{3}}$ to $R_{i}$.
Step PTA 4: $R_{i}\overset{M_{A_{4}}}{⟶}{\mathrm{tag}}_{i}:\langle M_{A_{4}}=\left\{V_{4},T_{4},Z_{T}\right\}\rangle$
  Upon receiving $M_{A_{3}}$, $R_{i}$ checks freshness of the timestamp $(T_{4}-T_{3})\leq \Delta T$. $R_{i}$ computes $h(R_{i}∥N_{r}∥K_{rs})$ and verifies its equality with the received $V_{3}$. Upon success, $R_{i}$ sends $M_{A_{4}}$ to $tag_{i}$. Otherwise, $R_{i}$ terminates the session.
Step PTA 5: Upon receiving $M_{A_{4}}$, $tag_{i}$ first checks freshness of the timestamp and upon success verifies the message $V4^{*}\overset{?}{=}h(K_{ts}∥ID_{T_{i}}∥N_{t})$. Then $tag_{i}$ computes and updates $AID_{T_{i}\left(new\right)}=\left(Z_{T}⊕K_{Ts}\right)$, $AID_{T_{i}}=AID_{T_{i}\left(new\right)}$ and saves the information for the next authentication process.

## 3. Security Analysis

In this section, the security analysis of the proposed protocol under the adversarial model briefed in Section 1.2 is performed. The task is accomplished by formal analysis under Burrows Abadi-Needham (BAN) logic, and informal security features are explained. Moreover, the robustness of the proposed protocol is also analyzed through the automated tool, ProVerif—a widely accepted simulation tool for verification of the security of authentication protocols [35,36,37,38,39,40,41,42,43].

### 3.1. BAN Logic-Based Formal Security Analysis

BAN logic consists of a set of rules that can be used to analyzed information exchange protocols. It specifically determines if the information exchanged in a protocol is resistant against eavesdropping and is trustworthy and secured. The mutual authentication of the proposed protocol has been checked using the BAN logic [44]. Different rules of BAN logic, including idealized form, assumptions, and proofs, are shown in Table 3.

To analyze the security of a protocol using BAN logic, different goals have to be determined. In the case of the proposed protocol, eight different goals have been determined based on BAN logic. These goals are shown in the following list. 2

•  Goal 1: $R_{i}|\equiv tag\overset{AID_{T}}{⟷}$ Ri•  Goal 5: $R_{i}|\equiv S_{j}\overset{AID_{T}}{⟷}$ Ri•  Goal 2: $R_{i}\left|\equiv tag\right|\equiv tag\overset{AID_{T}}{⟷}$ Ri    •  Goal 6: $Ri|\equiv S_{j}|\equiv S_{j}\overset{AID_{T}}{⟷}$ Ri•  Goal 3: $S_{j}|\equiv R_{i}\overset{AID_{t}}{⟷}$ Sj•  Goal 7: $tag|\equiv R_{i}\overset{AID_{T}}{⟷}$ tag•  Goal 4: $S_{j}\left|\equiv R_{i}\right|\equiv R_{i}\overset{AID_{T}}{⟷}$ Sj•  Goal 8: $tag|\equiv R_{i}|\equiv R_{i}\overset{AID_{T}}{⟷}$ tag.

To achieve the goals listed above, the security analysis using BAN logic has been divided into three parts. Part1 shows the idealized form of the protocol and is proved in Part3, whereas Part2 uses assumptions to analyzed the proposed protocol.

**Part1:** The idealized form for the proposed protocol has been discussed as follows:

- M1: tag→ Ri: $AID_{T}$,$N_{x}:<N_{t}{>}_{K_{ts}},V1,T1$
- M2: $R_{i}\to$ Sj: M1,$N_{y}:<N_{r}{>}_{K_{rs}},R_{i},V2,T2,$
- M3: $S_{j}\to$ Ri: V3,V4,$Z_{t}:<AIDT_{i}{>}_{K_{ts}}^{*},T3$
- M4: $R_{i}\to tag:V4,T4,Z_{t}:<AIDT_{i}{>}_{K_{ts}}$.

**Part2:** The assumptions used for analyzing the proposed protocol using BAN logic are shown below: 2

•  A1: $tag|\equiv \#(N_{t})$•  A6: $S_{j}|\equiv R_{i}\Rightarrow N_{r}$•  A2: $R_{i}|\equiv \#\left(N_{r}\right)$•  A7: $S_{j}|\equiv tag\Rightarrow N_{t}$•  A3: $S_{j}|\equiv \#\left(AIDTi\right)\left(r_{i}\right)$   •  A8: $tag|\equiv S_{j}\Rightarrow r_{i}$•  A4: $R_{i}|\equiv S_{j}\Rightarrow r_{i}$•  A9: $tag|\equiv R_{i}\Rightarrow N_{r}$.•  A5: $R_{i}|\equiv tag\Rightarrow N_{t}$


**Part 3:** Analysis of Idealized form of the proposed protocol that has been derived on the basis of BAN logic assumptions and rules is described as follows:

M1: tag→ Ri: $AIDT_{i}$,$N_{x}:<N_{t}{>}_{K_{ts}}$, T1 is time-stamp of tag. Using the seeing rule, the following can be achieved:

- S1: $R_{i}⊲AIDT_{i},SID,N_{x}:<N_{t}{>}_{K_{ts}},T1$.

According to the message-meaning rule and S1, the following can be obtained:

- S2: $R_{i}\left|\equiv tag\right|∼N_{t}$.

Using the freshness-conjuncatenation rule and S2 will achieve the following:

- S3: $R_{i}\left|\equiv tag\right|\equiv N_{t}$.

Using the jurisdiction rule and S3, the following can be achieved:

- S4: $R_{i}|\equiv N_{t}$.

Using S4 and the session key rule, the following can be achieved:

- S5: $R_{i}|\equiv tag\overset{AIDT_{i}}{⟷}$ Ri **(Goal 1)**.

Using the nonce-verification rule, the following is obtained:

- S6: $R_{i}\left|\equiv tag\right|\equiv tag\overset{AIDT_{i}}{⟷}$ Ri **(Goal 2)**.

M2: $R_{i}\to S_{j}:M1,N_{y}:<N_{r}{>}_{K_{rs}},T2,V2$, whereas T2 is time-stamp of $R_{i}$.

By using the seeing rule, we achieve:

- S7: $S_{j}⊲M1,N_{y}:<N_{r}{>}_{K_{rs}},T2,V2$.

By the message-meaning rule and S7, the following can be achieved:

- S8: $S_{j}\left|\equiv R_{i}\right|∼N_{r}$.

By the freshness-conjuncatenation rule and S8, the following can be computed:

- S9: $S_{j}\left|\equiv R_{i}\right|\equiv N_{r}$.

By applying the jurisdiction rule and S9, the following can be obtained:

- S10: $S_{j}|\equiv N_{r}$.

Using the S10 and the SK rule, the following can achieved:

- S11: $S_{j}|\equiv R_{i}\overset{AIDT_{i}}{⟷}$ Sj **(Goal 3)**.

Using the nonce-verification rule and S11, the following can be achieved:

- S12: $S_{j}\left|\equiv R_{i}\right|\equiv R_{i}\overset{AIDT_{i}}{⟷}$ Sj. **(Goal 4)**.

M3: $S_{j}\to$ Ri: $V3,V4,Zt<AIDT_{i_{new}}{>}_{K_{ts}}^{*},T3$, T3 is time-stamp of $S_{j}$.

By the seeing-rule, the following can be achieved:

- S13: $R_{i}⊲V3,V4,Zt<AIDT_{i_{new}}{>}_{K_{ts}}^{*},T3$.

By the message-meaning rule and S13, the following can be obtained:

- S14: $R_{i}\left|\equiv S_{j}\right|∼AIDT_{i_{new}}$.

By S14 and the freshness-conjuncatenation rule, the following can achieved:

- S15: $R_{i}\left|\equiv S_{j}\right|\equiv AIDT_{i_{new}}$.

By the assumption S15 and jurisdiction rule, the following can be achieved:

- S16: $R_{i}|\equiv AIDT_{i_{new}}$.

Using S16 and the session-key rule, the following can be achieved:

- S17: $R_{i}|\equiv S_{j}\overset{AIDT_{i_{new}}}{⟷}$ Ri. **(Goal 5)**.

Applying nonce-verification rule, the following can be computed:

- S18: $R_{i}\left|\equiv S_{j}\right|\equiv S_{j}\overset{AIDT_{i_{new}}}{⟷}$ Ri. **(Goal 6)**.

M4: $R_{i}\to tag:V4,Zt<AIDT_{i_{new}}{>}_{K_{ts}},T4$, T4 is timestamp of $R_{i}$.

Using the seeing rule, the following can be computed:

- S19: $tag⊲V4,Zt<AIDT_{i_{new}}\geq {>}_{ts},T4$.

Using the message-meaning rule and S19, the following is achieved:

- S20: $tag|\equiv R_{i}|∼AIDT_{i_{new}}^{\prime }$.

Using S20 and the freshness-conjuncatenation rule, the following can be obtained:

- S21: $tag|\equiv R_{i}|\equiv AIDT_{i_{new}}$.

Using the jurisdiction rule and S21, the following can be achieved:

- S22: $tag|\equiv AIDT_{i_{new}}$.

Using the session-key rule, the following can be obtained:

- S23: $tag|\equiv R_{i}\overset{AIDT_{i_{new}}}{⟷}tag$ **(Goal 7)**.

Finally, using the nonce-verification rule, the following can be achieved, which is also the final goal of the proposed protocol:

- S24: $tag|\equiv R_{i}|\equiv R_{i}\overset{AIDT_{i_{new}}}{⟷}tag$ **(Goal 8)**.

Consequently, using the BAN logic, it has been shown that tag, $R_{i}$, and $S_{j}$ achieve mutual authentication successfully and securely attain the session key agreement.

### 3.2. Security Analysis with ProVerif

**Figure 6:**
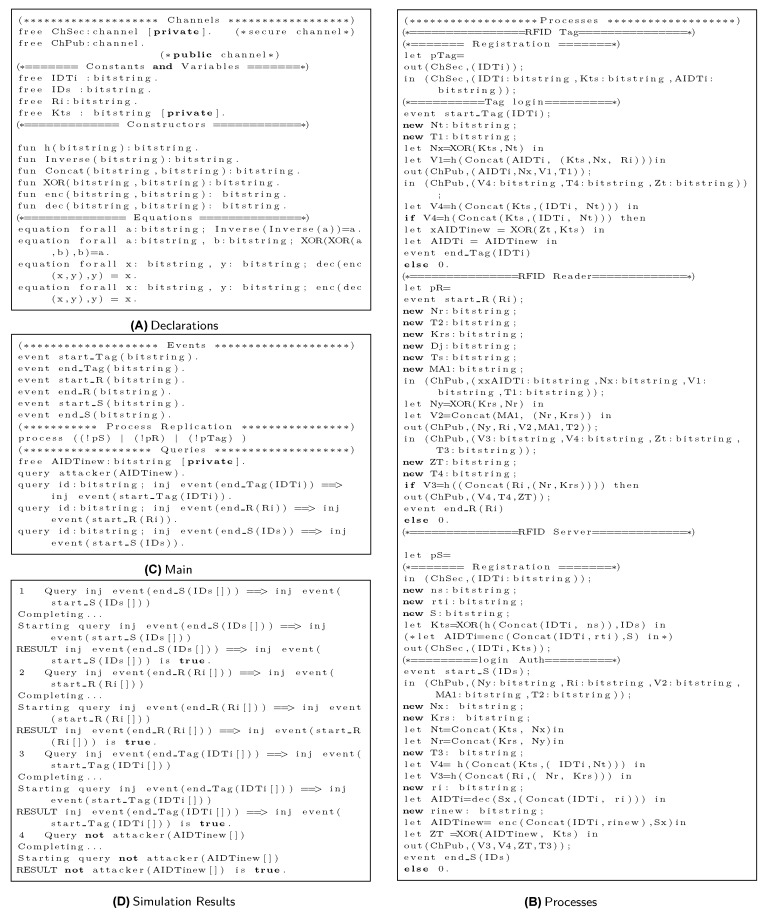
ProVerif Simulation.

Based on applied π calculus, ProVerif uses automated reasoning to test the security features of authentication protocols. Specifically, ProVerif can verify the reachability, correspondence, and observational equivalence, as well as secrecy properties. ProVerif supports primitive cryptographic operations [45], including MAC, digital signatures, encryption/decryption, elliptic curve operations, hash, and other functions [46]. The steps of the proposed scheme, as illustrated in Section 2 and shown in Figure 4 and Figure 5, are simulated in ProVerif. The formal security validation model of ProVerif consists of three phases: (1) Declaration, as coded in Figure 6A, declares the constants, names, variablesm, and cryptographic function, (2) Process part, as shown in Figure 6B, defines the three processes, each for tag, Reader, and Server, and (3) Main, as implemented in Figure 6C, simulates the actual protocol.

Simulation of three processes executed in parallel is performed, along with six events to validate the reachability properties of three processes. Finally, four queries are implemented. The results are shown in Figure 6D. Based on the above description of results 1, 2, and 3, all three original processes of the proposed protocol successfully started and terminated. Result 4 shows that the session tag identity $AID_{T_{i}}$ is safe from any adversary attack. Therefore, the proposed protocol possesses correctness and provides tag secrecy.

### 3.3. Informal Security Analysis

The proposed protocol for RFID System is analyzed for security loopholes against the known attacks in the following subsections.

#### 3.3.1. Mutual Authentication Between Tag And Server

The RFID Server authenticates RFID tag by verifying a one time alias $AID_{T_{i}}$ and $V_{1}\;=\;h(ID_{T}\left|\right|K_{ts}\left||N|\right|R_{i})$ in the message $M_{1}$. Only a legitimate RFID tag can form a valid request message $M_{1}$, including both these parameters, as valid $AID_{T_{i}}$ is only known to legal tag; moreover, $ID_{T}$, $K_{ts}$ are known to the legal tag only. On other side, the RFID tag can authenticate the legitimacy of the Server using parameters $V_{4}$ and message $M_{3}$ in $M_{4}$. This way, the proposed protocol achieves mutual authentication property.

#### 3.3.2. Anonymity

One of the basic principles of security is that an authentication protocol must not reveal the identity information of any participant (user or device) to an adversary. Anonymity is an essential factor of a secure protocol. A secure scheme guards the personal information of a user so that an adversary or intruder cannot access any information that may lead to a security breach of the system. In the proposed protocol, strong anonymity has been achieved. In the registration phase, the RFID tag registered itself with the Server *S* through RFID-Reader using a secure channel, $M=\{ID_{T_{i}},K_{ts},AID\}$.

In the login and authentication phase of the proposed protocol, message $M_{A_{1}}=\left\{AID_{T_{i}},N_{x},V_{1},T_{1}\right\}$ has been sent to the Server *S* using public channel. Here, if an adversary gets the message $M_{1}$, the adversary still cannot know the identity of the RFID tag because $AID_{T_{i}}$ is a one-time alias identity of the tag. The original identity is kept encrypted in $AID_{T_{i}}$ and can only be decrypted by the Server using a shared secret Key $K_{ts}$. Thus, an adversary cannot reveal the RFID tag’s actual identity, hence achieving anonymity for the proposed protocol.

#### 3.3.3. Traceability

A genuinely secure protocol must not reveal any identifying information of the participants to an illegitimate user. The identifying information may lead to the traceability of the RFID tag. The proposed protocol does not reveal any login information of the current of or any previous sessions that lead to a security attack on the RFID system. It is achieved through the use of different random numbers at different levels, like $N_{t},N_{r},r_{i}$. Furthermore, a new one-time-alias identity for the RFID tag $AID_{Ti}$ has been use, making it impossible for an adversary to guess any random number and launch an attack on the RFID system. Consequently, it can be been claimed that the proposed protocol makes the RFID tag untraceable.

#### 3.3.4. Backward/Forward Secrecy

It is essential for security protocols that the information transmitted in a session is not compromised, as well as traced or used by an adversary to create vulnerabilities in the current, previous, or future authentication session between the RFID tag and RFID Server *S*. In the proposed protocol, even if the identity $ID_{T}$ or alias identity are lost, it does not affect previous or next sessions. It is ensured through the use of encrypted $AID_{Ti}$, which is updated in every new session. In this way, the proposed protocol for the RFID System guarantees backward and forward secrecy.

#### 3.3.5. Scalability

In the proposed protocol for the RFID System, the RFID Server *S* does not perform an exhaustive process to authenticate any RFID-tag. Instead, the RFID-Server *S* processes $AID_{Ti}$ to validate the RFID tag and responds quickly to the RFID tag. This makes the proposed protocol more scalable.

#### 3.3.6. Collision Attack

If RFID-tags share the same credentials for authentication to access the RFID Server, the protocol may be left vulnerable to a collision attack. In the proposed protocol, every RFID tag uses different parameters, i.e., $\left\{N_{y},R_{i},V_{2},M_{A_{1}}\right\}$, for authentication that makes it impossible for collision attack to take place.

#### 3.3.7. DoS Attack

The protocol is not based on any random key that is responsible for authentication or verification of the RFID tag; rather, it is based on $AID_{Ti}$ that is well encrypted and updated for every transaction. Therefore, the proposed scheme resists any DoS attack.

#### 3.3.8. Replay Attacks

In a replay attack, the attacker may delay or repeat the transmitted information for authentication with the Server *S*. The proposed protocol for RFID systems has three participants: tag, Reader, and Server. For authentication, four messages are exchanged, i.e, $\left\{M_{1},M_{2},M_{3},M_{4}\right\}$, using a public channel. Having access to the messages, an adversary *A* may attempt to launch a replay attack. However, this attempt will fail as every message is sent with a fresh time-stamp *T*. In case the time-stamp is invalid, the adversary *A* request will be rejected each time. Furthermore, if an adversary *A* cannot compute other parameters of the message, the adversary still cannot launch the attack as all message parameters are updated for every new session by the participants of RFID System. Therefore, the proposed protocol for RFID systems is resistant to replay attack.

#### 3.3.9. Location Tracking Attack

As the real identity of the RFID tag is not sent directly in the message for authentication between the RFID-tag and Server S, it has been sent in an encrypted form that only the Server can decrypt using its secret key. Moreover, the messages exchanged among the participants are constantly updated in every new session that provides unpredictability. Hence, an adversary cannot find the location and any attempt of finding the location will ultimately fail.

#### 3.3.10. Impersonation Attacks (Forgery Attacks)

An adversary *A* may intercept the messages of the previous legitimate RFID tag and modify that for authentication with the RFID Server *S*. In this case, the adversary *A* needs to make a valid message request that includes different parameters, like $N_{y},R_{i},V_{2},M_{A_{1}},AID_{Ti}$. To do so, the adversary *A* must compute $AID_{Ti}$ that is well encrypted and impossible to be computed or forged. Moreover, the adversary *A* also needs different other parameters and timestamps to put a valid request for authentication as a legitimate RFID tag. It is impossible for the adversary *A* without knowing the actual parameters of the Message used for authentication, hence leaving the adversary *A* unable to prove its legitimacy as an RFID tag to the RFID Server *S*. Reluctantly, the proposed protocol for RFID System resists any forgery attack.

#### 3.3.11. Stolen-Verifier Attacks

The proposed protocol resists stolen-verifier-attack. All the verification and validation keys are stored encrypted in the RFID Database Server *S*. If the data and keys are stolen from the RFID Database Server *S*, still the adversary *A* cannot decrypt and extract them. Also, the adversary *A* cannot alter or modify the original data saved in the RFID Database Server *S*. Hence, the proposed protocol resists any stolen-verifier attack.

## 4. Comparative Analysis

This section presents a comprehensive comparative analysis of the proposed protocol with the existing protocols [3,21,23,30,31], as these schemes are based on lightweight symmetric key primitives. Hence, they are eligible for a fair comparison with the proposed scheme. Firstly, the proposed protocol is compared with the existing protocols in terms of security requirements. Secondly, a comparison of the proposed protocol with existing protocols based on computation cost (running time or execution time) is given, and thirdly, a comparison based on communication cost is presented. Furthermore, the proposed protocol is analyzed for storage complexity. Please note that we have selected the schemes based on lightweight symmetric key primitives and also that have been published recently. Each of these comparisons has been elaborated in the following subsections, one-by-one.

### 4.1. Security Requirements

Security requirements are the features expected from an authentication protocol. Every authentication protocol must be able to ensure these features or requirements. By these requirements, the proposed protocol is compared with the existing protocols. Following is the list of features/requirements considered for comparative analysis. 2

•  SR1: Mutual authentication.•  SR7: DoS attacks.•  SR2: Tag untraceability.•  SR8: Replay attacks.•  SR3: Tag anonymity.•  SR9: Location tracking attack.•  SR4: Backward/Forward secrecy.    •  SR10: Forgery attack.•  SR5: Scalability.•  SR11: Stolen-verifier attacks.•  SR6: Collision attacks.


Table 4 shows the security requirements comparison of the proposed protocol with existing symmetric key-based protocols [3,21,23,30,31].

The insecurities of the existing schemes [3,21,23,30,31] are well defined in Section 1 and are replicated in Table 4. The security requirements in Table 4 show that only the proposed protocol provides all security features.

### 4.2. Computation Cost Analysis

This section describes the computation cost analysis of the proposed protocol with existing related protocols [3,21,23,30,31]. For analysis purposes, the following notations are introduced:

- CC: Computation cost;
- $T_{h}$: CC of single hash function;
- $T_{se}$: CC of symmetric encryption/decryption.

**Figure 7:**
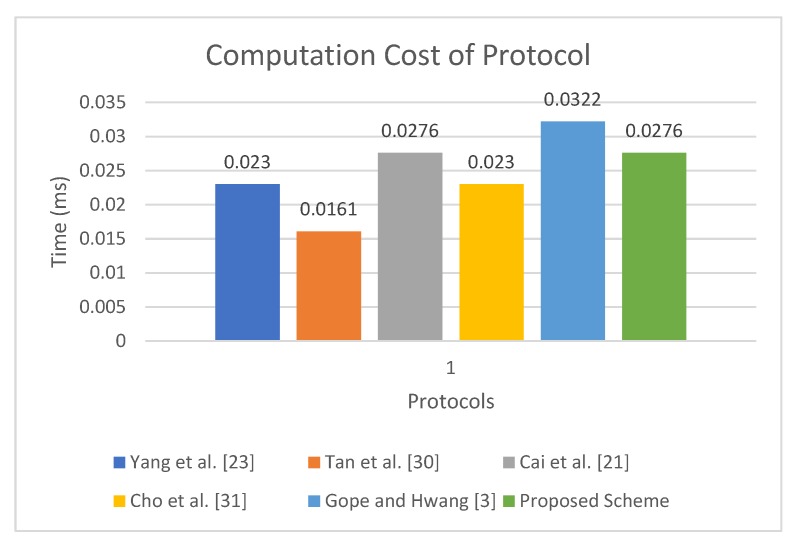
Running Time of Proposed Scheme.

Table 5 shows the computation cost analysis. The protocol presented in [23] incurs $2T_{h}$, $3T_{h}$, and $5T_{h}$, for each tag, Reader and Server, respectively, making its total computation cost $10T_{h}$. Similarly, the computation cost of protocol presented in [30] is $2T_{h}$, $2T_{h}$, and $3T_{h}$, respectively, for each participant, totaling it to $7T_{h}$. The protocol presented in [21] requires $4T_{h}$, $2T_{h}$, and $6T_{h}$ for each tag, Reader, and Server, respectively, totaling it to $12T_{h}$. The computation cost of the protocol of Gope and Hwang [3] is $5T_{h}$, $2T_{h}$, and $7T_{h}$, respectively, for each participant totaling it to $14T_{h}$. In comparison, the tag in proposed protocol uses $2T_{h}$, the Reader uses $2T_{h}$, and the Server requires $4T_{h}+2T_{se}$, so in total the computation cost of the proposed protocol is equal to $8T_{h}+2T_{se}$. Considering the experiment of Kilinc and Yanik [47], the computation time of $T_{h}$ is 0.0023 ms, whereas the computation time to calculate $T_{se}$ is 0.0046 ms. The experiment was performed on a Ubuntu system with an Intel dual-core Pentium processor with specifications, including 2.20 GHz, 2048 MB processor and Ram, respectively. The total computation time of the proposed protocol is 0.0276 ms, whereas the total cost of the protocol presented in [23] is 0.0230 ms, the cost of protocol in [30] is approximately 0.0161 ms, the cost of the proposal in [3] is 0.0322 ms, and the proposal in [21] takes a total of 0.0276 ms. Although the proposed protocols incur a slightly higher computation cost as compared with [23,30], it provides less computation cost when compared with the baseline [3] and provides the same computation cost as compared to the protocol presented in [21]. Moreover, the proposed protocol is the only protocol that provides resistance against all known attacks. The results presented in Table 5 are visualized in Figure 7.

### 4.3. Communication and Storage Cost Analysis

Communication cost is presented in terms of the total number of messages exchanged and total number of bits exchanged during one transaction of the protocol. In the proposed protocol, $tag_{i}$ transmits four parameters in $M_{1}$ to $R_{i}$ carrying 416bits and receives 384bits from $R_{i}$. Similarly, $R_{i}$ transmits 736bits and receives 416bits from *S*, while *S* transmits 416bits and receives 736bits. The communication cost comparison of the proposed protocol with other existing protocols is presented in Table 6. The storage cost is represented by length Value *L*, the proposed protocol uses SHA-1 hash function to implement h(.); for simplicity, each of the length values is considered as 160-bit long. In the proposed scheme, each tag stores $ID_{T_{i}},K_{ts},AID$ parameters. Therefore, the cost of storage in the tag is 3L, whereas on the Server side, $ID_{T_{i}},K_{ts},AID_{new},AID_{old}$ are being stored; hence, the storage cost on the Server side is 4L per tag.

**Figure 8:**
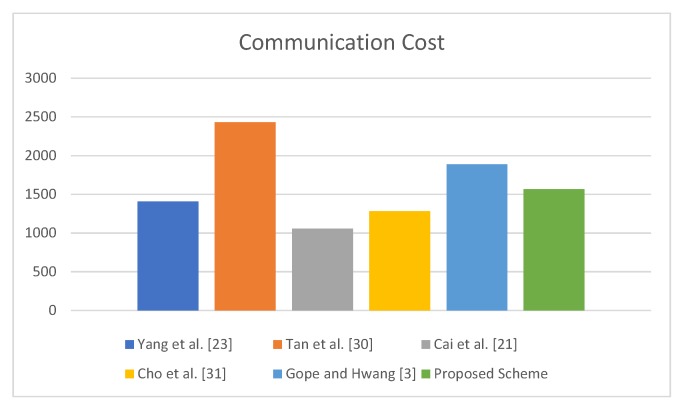
Communication Cost.

The proposed protocol incurs less communication cost as compared with the protocols of [3,30], whereas it has more communication cost when compared with others [21,23,31]. However, only the proposed protocol provides required security. The results presented in Table 6 are visualized in Figure 8.

Table 6 indicates that the proposed protocol is more efficient than the baseline protocol in terms of communication cost. Specifically, the proposed protocol not only overcomes the security flaws of the baseline protocol but also achieves 16.94% efficiency in terms of the number of bits exchanged during one transaction of the protocol.

## 5. Conclusions

In this article, cryptanalysis of a recent authentication protocol by Gope and Hwang has been presented, and it has been proved that their protocol has some weaknesses against collision, stolen verifier, and DoS attacks. An improved scheme using only lightweight primitives is proposed to resist all known attacks. The security of the proposed scheme has been thoroughly analyzed informally, as well as formally, using BAN logic. Moreover, the scheme is simulated in automated applied π calculus-based tool ProVerif. The simulation also backs the formal and informal security analysis. Although the proposed scheme incurs some extra computation and communication cost as compared with some existing related protocols, only the proposed protocol resists all known attacks and is more suitable for practical IoT-based scenarios.

## Figures and Tables

**Table 1:** RFID-tag features.

Features	Passive Tags	Active Tags
Data Storage	128 bytes	128 bytes
tag Power	Energy transferred through Radio Frequency from Reader	Internal source to tag
tag Battery	No	Yes
Availability of Source Power	Only in range of Radar	Continuous
Signal Strength required to tag	Very High	Very Low
Range	Upto 3–5 M	Upto 100 M
Multiple tag Reading	less then thousand tags within 3 M of Reader range	More then 1000 tags recognized upto 100 mph

**Table 2:** Notation Guide.

Notations	Description
*T*	RFID-tag
*R*	Reader Device
*S*	Database Server System
$ID_{T_{i}}$	ith tag identity
$AID_{T}$	One-time tag alias identity
SID	Shadow identity
$R_{j}$	jth Reader identity
$N_{t}$	tag Random number
$N_{r}$	Reader Random number
$K_{ts}$	Shared key of Server and tag
$K_{emg}$	Shared emergency key of Server and tag
$K_{rs}$	Server and Reader shared secret key
$Tr_{seq}$	Track sequence number (used by both S and T)
$r_{j}$	Randomly derived from Shadow-ID and Emergency Key
h(.)	Hash function
⊕	The exclusive XOR operation
||	concatenation

**Table 3:** BAN logic Notations.

Notations	Description
P|≡X	P believes that X
P⊲X	P sees that X
P|∼X	P once said X
P⇒X	P have total jurisdiction on X
#(X)	X is updated and fresh
(X,Y)	X, Y is component of formula(X,Y)
$<X{>}_{Y}$	X is combine with Y
${\left(X\right)}_{K}$	Hash of message X using a key K
$P\overset{K}{⟷}Q$	P and Q share key K for communication
$AIDT_{i}$	$AIDT_{i}$ is one time session key
$\frac{P|\equiv P\overset{K}{⟷}Q.p⊲<X{>}_{K}}{P|\equiv Q|∼X}$	Message-Meaning rule
$\frac{P|\equiv \#(X)}{P|\equiv \#(X,Y)}$	Freshness-conjuncatenation rule
$\frac{P|\equiv \#(X),P|\equiv Q|∼X}{P|\equiv Q|\equiv X}$	Nonce-verification rule
$\frac{P|\equiv Q\Rightarrow X,P|\equiv Q|\equiv X}{P|\equiv X}$	Jurisdiction rule
P|≡X	P believes X

**Table 4:** Security requirements table.

Requirements	Yang et al. [23]	Tan et al. [30]	Cai et al. [21]	Cho et al. [31]	Gope et al. [3]	Proposed Scheme
SR1	×	×	√	√	√	√
SR2	×	×	×	√	√	√
SR3	×	×	√	×	√	√
SR4	×	√	×	√	√	√
SR5	×	×	×	×	√	√
SR6	×	×	×	√	×	√
SR7	√	×	√	√	×	√
SR8	√	√	√	√	√	√
SR9	√	√	√	√	√	√
SR10	√	√	√	√	√	√
SR11	√	√	√	√	×	√

√: Yes provides, ×: Does not provide.

**Table 5:** Comparison of computation cost and running time.

Computation Cost	Yang et al. [23]	Tan et al. [30]	Cai et al. [21]	Cho et al. [31]	Gope and Hwang [3]	Proposed Scheme
$CC_{tag}$	2$T_{h}$	2$T_{h}$	4$T_{h}$	3$T_{h}$	5$T_{h}$	2$T_{h}$
$CC_{R_{i}}$	3$T_{h}$	2$T_{h}$	2$T_{h}$	2$T_{h}$	2$T_{h}$	2$T_{h}$
$CC_{S}$	5$T_{h}$	3$T_{h}$	6$T_{h}$	5$T_{h}$	7$T_{h}$	4$T_{h}+2T_{se}$
$CC_{Total}$	10$T_{h}$	7$T_{h}$	12$T_{h}$	10$T_{h}$	14$T_{h}$	8$T_{h}+2T_{se}$
$CC_{Time}$	0.023 ms	0.0161 ms	0.0276 ms	0.023 ms	0.0322 ms	0.0276 ms

**Table 6:** Communication Cost of Proposed and other Protocols.

Schemes	tag	Reader	Server	Total Bits	Messages
Yang et al. [23]	256	512	640	1408	5
Tan et al. [30]	896	768	768	2432	4
Cai et al. [21]	256	544	256	1056	5
Cho et al. [31]	512	512	256	1280	5
Gope and Hwang [3]	416	1180	288	1888	4
Proposed Protocol	416	736	416	1568	4
